# Electroacupuncture Alleviates Pain-Related Emotion by Upregulating the Expression of NPS and Its Receptor NPSR in the Anterior Cingulate Cortex and Hypothalamus

**DOI:** 10.1155/2020/8630368

**Published:** 2020-02-10

**Authors:** Zitong Xu, JunFan Fang, Xuaner Xiang, HaiJu Sun, SiSi Wang, Jianqiao Fang, Junying Du

**Affiliations:** Department of Neurobiology and Acupuncture Research, the Third Clinical Medical College, Zhejiang Chinese Medical University, Key Laboratory of Acupuncture and Neurology of Zhejiang Province, Hangzhou 310053, China

## Abstract

**Objective:**

Electroacupuncture (EA) is reported effective in alleviating pain-related emotion; however, the underlying mechanism of its effects still needs to be elucidated. The NPS-NPSR system has been validated for the involvement in the modulation of analgesia and emotional behavior. Here, we aimed to investigate the role of the NPS-NPSR system in the anterior cingulate cortex (ACC), hypothalamus, and central amygdala (CeA) in the use of EA to relieve affective pain modeled by complete Freund's adjuvant- (CFA-) evoked conditioned place aversion (C-CPA). *Materials and Methods*. CFA injection combined with a CPA paradigm was introduced to establish the C-CPA model, and the elevated O-maze (EOM) was used to test the behavioral changes after model establishment. We further explored the expression of NPS and NPSR at the protein and gene levels in the brain regions of interest by immunofluorescence staining and quantitative real-time PCR.

**Results:**

We observed that EA stimulation delivered to the bilateral Zusanli (ST36) and Kunlun (BL60) acupoints remarkably inhibited sensory pain, pain-evoked place aversion, and anxiety-like behavior. The current study showed that EA significantly enhanced the protein expression of this peptide system in the ACC and hypothalamus, while the elevated expression of NPSR protein alone was just confined to the affected side in the CeA. Moreover, EA remarkably upregulated the mRNA expression of NPS in CeA, ACC, and hypothalamus and NPSR mRNA in the hypothalamus and CeA.

**Conclusions:**

These data suggest the effectiveness of EA in alleviating affective pain, and these benefits may at least partially be attributable to the upregulation of the NPS-NPSR system in the ACC and hypothalamus.

## 1. Introduction

The concept of pain has been defined by the International Association for the Study of Pain as a distressing experience associated with actual or potential tissue damage and having sensory, emotional, cognitive, and social components [1], reflecting the multiple dimensions of pain. The limbic system includes a wide range of brain regions that are comprehensively interconnected in a complex paradigm to implement the processing and regulation of cognition, sensory perception, and the generation of emotional activities and affective motivation. These pain components occur through various pathways and interconnect with many brain areas like the anterior cingulate cortex (ACC) [2–4], hypothalamus [5, 6], and central amygdala (CeA) [7, 8].

The subdivisions of the ACC proved to be playing crucial roles in both cognitive and emotional processing of pain [9–12]. ACC stimulation induces ultrasonic vocalization and long-term fear memory in freely moving mice, indicating the close link between the ACC and negative emotion formation [13]. The hypothalamus is a component of the hypothalamic-pituitary-adrenal (HPA) axis and is involved in the regulation of depression, anxiety, and anorexia. The ventromedial hypothalamus (VMH) contributes to the processing of the affective dimension of pain [14] and the posterior hypothalamus (PH) is involved in the processing of cluster headache [15], showing evidence of the hypothalamus in the pain-related emotion. The amygdala plays a key role in delivering and organizing emotional information such as emotional learning, fear memory, and anxiety and depression [16–18]. The subregions of the CeA form as a collecting-processing position for pain and pain-related emotional information due to its anatomical features [19–21]. The CeA has been confirmed to decrease nocifensive and affective pain responses in an arthritic pain model [22], and pain-related synaptic plasticity occurs in the CeA under chronic pain [23]. The amygdala appears to be well documented in modulating pain in sensory and affective dimensions [24–26].

Neuropeptide S (NPS) was identified in 2002 as an endogenous ligand for the orphan G protein-coupled receptor (GPCR) GPR154 referred to as NPSR. Previous studies found that the robust expression of NPS precursor mRNA [27, 28] and NPSR mRNA [9, 29] dominated in large areas of the rats' brain by the in situ hybridization method, including the amygdala, cortex, and hypothalamus. NPS, binding to its receptor NPSR, increases intracellular calcium concentration and cAMP accumulation and has been validated for the involvement in the modulation of arousal, food intake, analgesia [30, 31], and emotional behavior [9, 32]. For instance, Jinushi et al. found that NPS mediated the activation of central noradrenergic neuronal activity to elicit the antinociceptive effect [30]. Intracerebroventricular (ICV) injection of NPS significantly evoked central antinociceptive effects during both phases of the formalin test by activating both A_1_ and A_2A_ receptors [33]. The combination of whole cell patch-clamp recordings and behavioral assays revealed exogenous NPS inhibited the synaptic activation of central nucleus (CeLC) neurons on a cluster of inhibitory intercalated (ITC) cells to perform like an anxiolytic agent [34]. Zoicas et al. extended the potent anxiolytic profile of NPS in reducing social fear and social avoidance [32]. On both the elevated plus maze and the open field, male adult Wistar rats showed reduced anxiety-like behavior after bilateral microinfusion of NPS (0.2 nmol/0.5 *μ*l) into the medial amygdala (MeA) [35]. By utilizing the selective NPSR antagonist, NPS was discovered as a potent agent in inhibiting pain-related emotional-affective behaviors through suppressing nociceptive processing in the amygdala [36] The studies referenced above highlighted the potential of the NPS/NPSR system in modulating pain-related emotions and behaviors.

Electroacupuncture (EA), whose efficacy has been widely recognized, is applied to treat different states of multiple acute and chronic pain diseases. Notably, it has been further validated that EA inhibited the affective dimension of pain on the basis of analgesia [37–39] Accordingly, proofs of the alleviation of pain and accompanied negative emotion performed by EA through the abovementioned brain areas bring us a new insight into the underlying mechanism [36, 40–43]. However, based on the limited number of studies, it remains to be adequately explored how EA promotes the relief of affective pain-related aspects by acting upon ACC, hypothalamus, and CeA.

Taken together, our data provide explicit prerequisites to hypothesize whether the NPS-NPSR system in the ACC, hypothalamus, and CeA mediates EA alleviation effects on pain-related emotion.

## 2. Materials and Methods

### 2.1. Animals

Male SD rats weighing 180–220 g at the start of the whole experiment were purchased from the Department of Animal Sciences of Zhejiang Chinese Medical University. All rats were cage-housed (*n* = 5) at a room temperature approximately 25 ± 2°C and on a 12 : 12 h light/dark cycle (lights on at 06 : 00) with a quiet outside environment and good ventilation and air filtration system. Water and chow pellets were available ad libitum. The animals were allowed to acclimate to the housing facilities for one week before the formal test, and all experimental procedures were performed in specially equipped rooms between 08 : 00 and 18 : 00. This study was approved by the Institutional Animal Care and Use Committee (IACUC-20180319-12), and all animal procedures conformed to the National Institutes of Health Guide for the Care and Use of Laboratory Animals. We have minimized the animals' suffering.

### 2.2. C-CPA Model Establishment and CPA Test

The conditioned place aversion (CPA) device was a shuttle box divided into two equal-sized compartments (length × width × height = 60 cm × 30 cm × 30 cm) separated by a movable partition. The background of the device was black, and one compartment, scented with cinnamon oil, had white triangle patterns, while the other compartment, scented with 2% acetic acid solution, had white circular patterns, and the two opposite walls of the partition were marked with patterns corresponding to the respective compartments. An infrared camera was attached to the top of the unit and connected to an external computer to record and analyze the time the rats stayed in the compartments on both sides during the free activity time (15 min) automatically. The apparatus was placed in a sound- and light-attenuated box under dim illumination, and white noise was played inside the enclosure.

#### 2.2.1. C-CPA Model Establishment

The acquisition of the model requires four distinct sessions:

1. *Acclimation*. On day 1, rats were individually placed on either side with the partition door removed and were allowed to explore the two compartments freely for 15 min to habituate to the apparatus.
2. *Preconditioning Session*. On day 2, the same trial was performed as on day 1, and the time spent in each compartment was measured as the baseline during the settled experimental recording time (12 min). Rats spending more than 70% of the total experimental time in one compartment were excluded. The compartment spent with a shorter residence time was defined as the nonpain-associated conditioned compartment and the other as the pain-associated conditioned compartment (CFA-paired compartment).
3. *CFA Conditioning Session*. On day 3, all rats were individually confined in the nonpain-associated conditioned compartment for 45 min with the partition embedded and then returned to the home cage. After 45 min, model rats were performed by subcutaneous injection of complete Freund's adjuvant (0.1 ml, Sigma, USA) into the left hind paw and were returned to the home cage again. After 6 hours, all rats were placed in the pain-associated conditioned compartment for 45 min. Rats in the control group received the same experimental procedures except the injection was replaced by the same volume of sterilized normal saline.
4. *Postconditioning Session.* On day 4, the experiment operation was conducted in the same way as the preconditioning session. The time spent in each compartment was measured. The time rats stayed in the pain-associated conditioned compartment on day 4 was less than the time spent in the same compartment on day 2; also, there was a significant difference indicating the successful establishment of the C-CPA model.

#### 2.2.2. CPA Test

The CPA test was performed on days 4 and 6. In this procedure, we tested the residence time in the two compartments during 12 min. Rats in the EA and sham EA groups received EA and sham EA treatment, respectively, before the test, and the whole observation was the same as the postconditioning session. Here, we obtained CPA scores and the magnitude of CPA scores. CPA scores were calculated by the time rats spent in the pain-associated conditioned compartment on day 4 or 6 minus that time on day 2, while the magnitude of CPA scores was just from the opposite count method.

### 2.3. Paw Withdrawal Thresholds

Von Frey hairs were applied to test the paw withdrawal thresholds (PWTs) to assess the inflammatory pain under mechanical stimuli [44]. Testing was performed from 14 : 00 to 17 : 00. The testing apparatus was plastic cages (20 cm × 20 cm × 15 cm) shaped as a rectangle. The apparatus was put on a wire mesh bottom (3 mm × 3 mm). Each rat was immobilized in a single plastic cage to adapt to the new environment for half an hour. The range of filament stiffness chosen in this experiment was as follows: 0.4, 0.6, 1.0, 1.4, 2.0, 4.0, 6.0, 8.0, 15.0, and 26.0 g. The filaments were applied vertically, and 4.0 g was the starting stiffness. The filament was applied perpendicularly to the central plantar surface of the left hind paw, avoiding the paw pads, and pressure was sustained for 6–8 s until an S-shape formed. When the starting stiffness could not induce positive responses (expressed as X or otherwise as O), another neighboring filament with greater stiffness was used. Identically, a weaker adjacent filament was chosen when an aversive response occurred. This procedure was repeated several times at 2 min intervals to achieve the first “OX” or “XO” combination, and then four additional filaments were applied in the same way to obtain a series of “O” or “X” results. When continuous positive or negative responses were observed within the range set, a stiffness of 26.00 g or 0.4 g, respectively, was recorded as the final value.

Data obtained were transformed into the 50 g threshold by using the following formula proposed by Chaplan: PWT(g) = (10^[Xf+*κδ*])^/10000, where Xf refers to the stiffness value of the final hair used, *k* refers to the tabular value for the pattern of the “OX” sequence, and *δ* means the average difference (in log units) between stimuli (here, 0.231). If the threshold calculated by this formula was higher than 26 g or lower than 0.4 g, then the value was recorded as 26 g or 0.4 g, respectively.

### 2.4. Elevated O-Maze

The apparatus consisting of a circular path (runway width 25 cm, diameter 100 cm) was placed 70 cm above the floor. Two opposing arms were protected by walls (closed area, height 30 cm), and the remaining ones were open arms without any protection. The length of the closed arms is equal to that of the open arms. The apparatus was placed on a dark surface in order to maintain control over lighting conditions during testing. Rats were placed in one of the closed arm areas of the apparatus with half of the body and the head towards the open arm. Behavioral data were recorded and assessed using SMART 3.0 software. The total observation time was set as 7 min with the first minute as the latency followed the 5 min experimental period. When one rat was finished, the apparatus was cleaned with 10% ethyl alcohol to eliminate the residual information (e.g., feces and odor). Total distance, distance traveled in the distinct arms, and time spent in the different arms were obtained.

### 2.5. EA Stimulation

The rats in the EA group were fastened with a designated soft cloth cover. The bilateral “Zusanli” (ST36, at the posterolateral aspect of the knee joint, approximately 5 mm below the humeral head) and “Kunlun” (BL60, approximately 10 mm above the prominence of the lateral malleolus of the hind limb) acupoints were inserted 5 mm in depth using stainless steel acupuncture needles with the size of 0.18 mm × 13 mm. The ipsilateral acupoints were connected to the HANS Acupuncture Point Nerve Stimulator (LH-202H; Huawei Co., Ltd., Beijing, China) through the output terminals. The treatment parameters were as follows: frequencies alternating at 2/100 Hz automatically with each frequency lasting 3 s and intensities of current ranging from 0.5 to 1.5 mA (starting at 0.5 mA, increasing 0.5 mA at a 10-minute interval, totaling 30 min). The stimulation was exerted once a day for 30 min before behavioral tests. For the observation of EA effects on C-CPA, the intervention of EA was performed on the conditioning session, the postconditioning session, the day after postconditioning, and the CPA test day. To investigate the EA effects on C-CPA-induced behavioral change, EA was administered on the conditioning session, the postconditioning session, the day after postconditioning, and the EOM test day.

**Figure 1:**
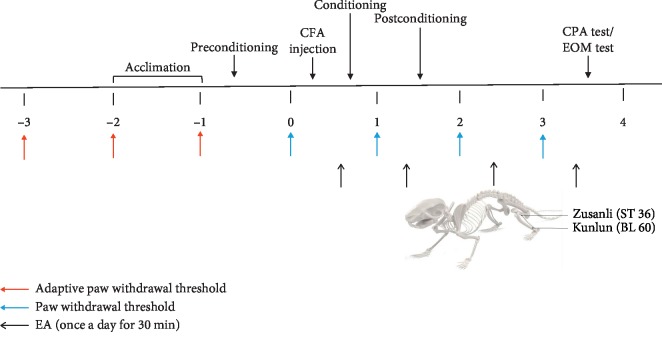
Timeline of the behavioral tests and the topographical diagram of the acupuncture stimulated points in rats. The observation of the effects of EA on the C-CPA model and on C-CPA-induced anxiety-like behaviors was conducted on two batches of animals.

The timeline of all the behavioral tests is shown in Figure 1.

### 2.6. NPS/NPSR Immunofluorescence Staining

Animals were deeply anesthetized with an overdose intraperitoneal injection of 1% sodium pentobarbital (60 mg/kg) followed by transcardial perfusion with 150 mL cold sterilized saline and 150 mL 4% paraformaldehyde in 0.1 M phosphate-buffered saline (PBS) and 150 ml paraformaldehyde by slow infusion. Brains and hypothalami were quickly harvested, postfixed in the same fixative overnight, and immersed successively in 15% and 30% sucrose solution at 4°C until the samples were dehydrated completely, after which they were coronally sectioned (30 *μ*m) on a cryostat (CryoStar NX50; Thermo Scientific, Britain) at −20°C, affixed to glass slides, and air-dried. The sections were blocked for 1 hour in 5% normal donkey serum blocking buffer (WB736984; Dawen Biotec) at 37°C, and the anti-NPS primary antibody (diluted 1 : 1000; n2412; Sigma) or NPSR primary antibody (diluted 1 : 750; orb158023; Biorbyt) was added overnight at 4°C. After washing three times (3 × 10 min) with TBST at room temperature, the slides were incubated with the Alexa Fluor 488-conjugated secondary antibody (diluted 1 : 400; ab150061; Abcam) for 1 hour at 37°C. Finally, the sections were washed with TBST six times (6 × 5 min), air-dried, and slide-covered with the anti-fluorescence quenching agent. Images were viewed and captured using a fluorescence microscope (Olympus IX71; Olympus, Tokyo, Japan). Image-Pro Plus software 7.0 (Media Cybernetics, USA) was used to analyze the relative expression of NPS and NPSR proteins.

### 2.7. Quantitative Real-Time PCR

The ACC, hypothalamus, and CeA were removed quickly under the same anesthesia with an overdose intraperitoneal injection of 1% sodium pentobarbital (60 mg/kg) followed by transcardial perfusion with 150 mL cold sterilized saline and preserved at −80°C. The mRNA expression of NPS and NPSR of rats was analyzed by quantitative real-time PCR (qPCR), using the CFX96™ real-time PCR detection system (Bio-Rad, USA) according to the manufacturer's instructions. Primer Premier 5.0 software (Premier, Canada) was used to design oligonucleotide primers specific for rat NPS, NPSR, and GAPDH (the internal control): NPS: 5′-TGTCGCTGTCCACAATGCAT-3′ and 5′-ATCAGATTTTCCAGACACCTTAGAAG-3′; NPSR: 5′-TGCAGGGAGCAAAGATCACA-3′ and 5′-AATCTGCATCTCATGCCTCTCA-3′; and GAPDH: 5′-TGCTGAGTATGTCGGAG-3′ and 5′-GTCTTCTGAGTGGCAGTGAT-3′. The primers selected in this experiment met the G-C content requirement with the melting temperature (Tm) at 60 and 70°C. All rats were deeply anesthetized with an overdose intraperitoneal injection of 1% sodium pentobarbital (60 mg/kg) followed by transcardial perfusion with 150 mL cold sterilized saline. The anterior cingulate cortex, hypothalamus, and central amygdala were removed and preserved at −80°C. Tissues were homogenized in (ml) Trizol solution (Invitrogen, France) and total RNA was extracted by the guanidinium thiocyanate method, after which reverse transcription was performed using the PrimeScript RT Reagent Kit with gDNA Eraser (TaKaRa, Japan). RNA was quantified by spectrophotometry. Water controls were included to ensure specificity. Experiments were performed in triplicates under the following PCR protocol: initial denaturation at 95°C for 30 min, 40 cycles of denaturation (95°C for 10 s), and then 30 s at 59°C (NPS mRNA)/56.0°C (NPSR mRNA). Melting curve analysis (65–95°C with 0.5°C/s) was included at the end of every run in order to ensure the homogeneity of the PCR product. The expression level of each candidate was normalized to glyceraldehyde phosphate dehydrogenase (GAPDH) in the same sample. The relative gene expression was determined by the 2^−△△CT^ method calculated from the relative standard curve.

### 2.8. Statistical Analysis

All data were expressed as the mean ± standard error (SE). The PWTs were analyzed using the independent-sample *t*-test. Data including CPA scores, magnitude of CPA scores, EOM, relative protein, and mRNA expression were analyzed using one-way ANOVA followed by the least-significant difference (LSD) post hoc test. The criterion for statistical significance was set as *P* < 0.05.

## 3. Results

### 3.1. CFA Injection Induced a Decrease in Paw Withdrawal Thresholds and C-CPA Model Establishment

**Figure 2:**
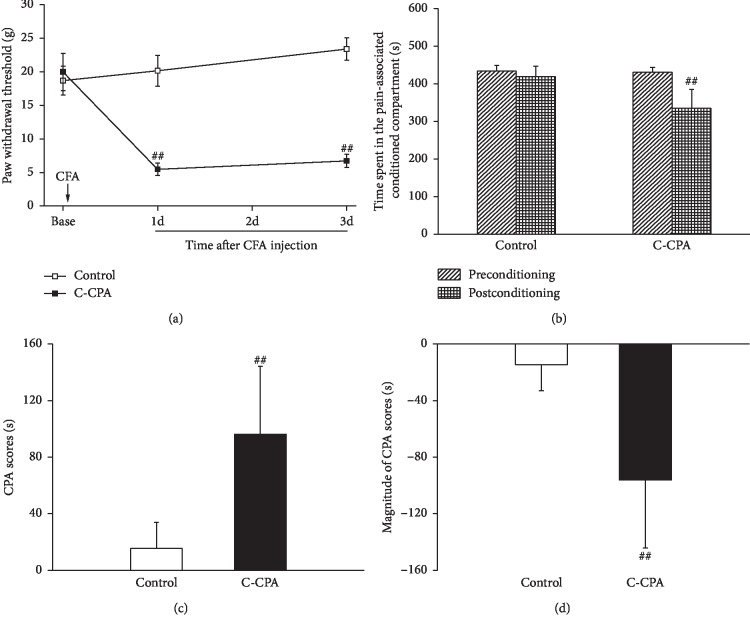
C-CPA model induced the decrease in paw withdrawal thresholds and the establishment of conditioned place aversion. (a) Paw withdrawal thresholds after CFA injection. (b) Time spent in the CFA-paired compartment on pre- and postconditioning days. (c) CPA scores on the postconditioning day. (d) Magnitude of CPA scores. Eleven rats in each group. ^##^*P* < 0.01, C-CPA vs. control.

As shown in Figure 2(a), PWTs in the C-CPA group were decreased significantly compared with those in the control group (5.47 ± 0.91 vs. 20.16 ± 2.28, *P* ≤ 0.001) 1 day and (6.72 ± 0.97 vs. 23.40 ± 1.67, *P* ≤ 0.001) 3 days after CFA injection, indicating the establishment of the inflammatory pain state. On this basis, we investigated the acute emotional change under this pain state induced by CFA injection; as shown in Figure 2(b), CFA injection decreased the time rats spent in the pain-associated conditioned compartment compared with the control group (335.36 ± 49.83 vs. 456.40 ± 16.50, *P*=0.035), manifesting the occurrence of conditioned place aversion. There was no difference between groups during the preconditioning session.

Figure 2(c) shows that the CFA-injected animals exhibited a much higher degree of conditioned place aversion than the normal animals (14.54 ± 18.37 vs. 96.21 ± 48.03, *P*=0.044), and the magnitude of CPA scores (−14.54 ± 18.37 vs. −96.21 ± 48.03, *P*=0.044, Figure 2(d)) informed clear sign on the establishment of the C-CPA model.

### 3.2. EA Ameliorated the Aversive Emotion Induced by the C-CPA Model

**Figure 3:**
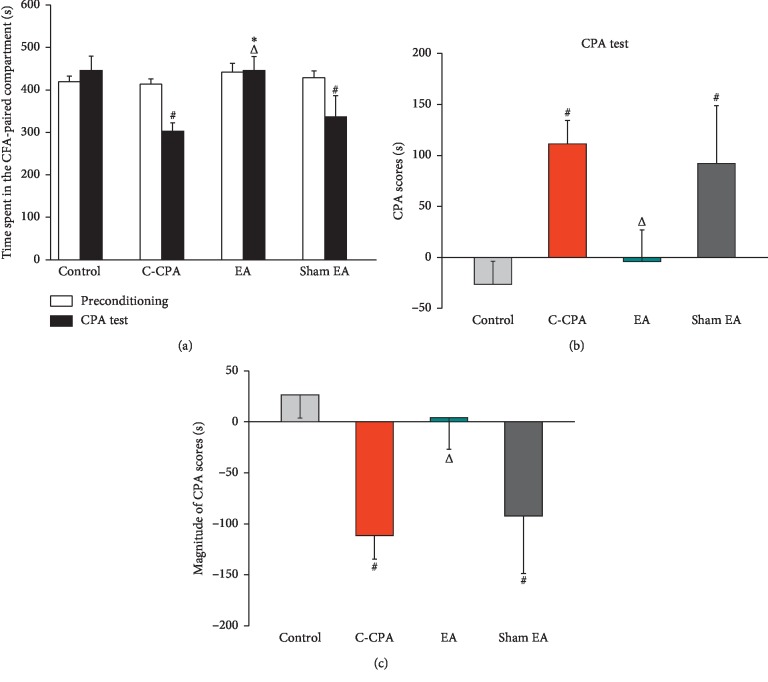
Effects of EA stimulation on aversive emotion induced by the C-CPA model on day 6. (a) Time spent in the CFA-paired compartment on the CPA test day in each group. (b) CPA scores on the CPA test day. (c) Magnitude of CPA scores in each group. Sixteen rats in each group. ^#^*P* < 0.05, C-CPA and sham EA vs. control; ^Δ^*P* < 0.05, EA vs. C-CPA; ^*∗*^*P* < 0.05, EA vs. sham EA.

To identify whether EA improves the aversive emotion of the C-CPA group, we observed the time spent in the pain-associated compartment and CPA scores after 4 consecutive days of EA stimulation. As shown in Figure 3(a), C-CPA induced decreased residence time in the CFA-paired compartment (302.38 ± 20.56 vs. 445.50 ± 33.67, *P*=0.012, Figure 3(a)), increased CPA scores (111.38 ± 23.07 vs. −26.45 ± 22.60, *P*=0.014, Figure 3(b)), and declined the magnitude of CPA scores (−111.38 ± 23.07 vs. 26.45 ± 22.60, *P*=0.014, Figure 3(c)) when compared with the control group. EA significantly abolished the negative emotion by increasing the time spent in the CFA-paired compartment (445.80 ± 33.18 vs. 302.38 ± 20.56, *P*=0.013, Figure 3(a)), attenuating the CPA scores (−4.13 ± 30.80 vs. 111.38 ± 23.0, *P*=0.041, Figure 3(b)), and elevating the magnitude of CPA scores (4.13 ± 30.80 vs. −111.38 ± 23.07, *P*=0.041, Figure 3(c)); sham EA did not affect these parameters as expected.

### 3.3. EA Alleviated the Anxiety-Like Behaviors Induced by C-CPA

**Figure 4:**
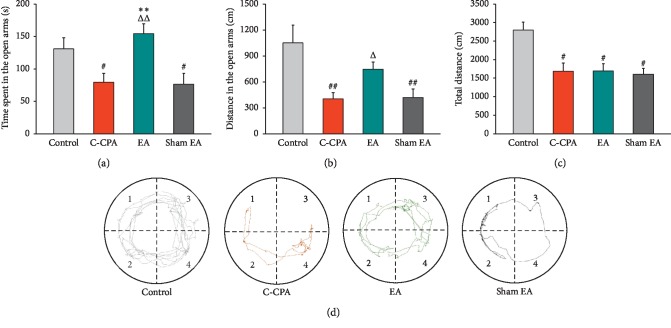
Effects of EA stimulation on anxiety-like behaviors induced by the C-CPA model on day 6. (a) Time spent in the open arms in each group. (b) Distance traveled in the open arms in each group. (c) Total distance traveled in the EOM apparatus in each group. (d) Representative pictures of animals' movement traces on the EOM apparatus in each group (Quadrants 1 and 4 represent the closed arms, while 2 and 3 the open arms). Sixteen rats in each group. ^#^*P* < 0.05 and ^##^*P* < 0.01, C-CPA, EA, and sham EA vs. control; ^Δ^*P* < 0.05 and ^ΔΔ^*P* < 0.01, EA vs. C-CPA; ^*∗∗*^*P* < 0.01, EA vs. sham EA.

To further investigate the interventional effects of EA on the C-CPA model, we observed behavioral changes through the elevated O-maze test. The results in Figures 4(a) and 4(b) revealed the C-CPA model caused a significant reduction on time spent (79.36 ± 13.98 vs. 130.94 ± 16.83, *P*=0.025, Figure 4(a)) and distance traveled (404.66 ± 72.27 vs. 1050.60 ± 207.22, *P*=0.001, Figure 4(b)) in the open arms compared with the control group. EA notably increased the residence time and traveled distance in the open arms (54.34 ± 15.30 vs. 79.36 ± 13.98, *P*=0.002, Figure 4(a), and 744.43 ± 83.43 vs. 404.66 ± 72.27, *P*=0.048, Figure 4(b), respectively), without affecting the total distance traveled in the apparatus. This emotional change could not be alleviated in the sham EA group (25.49 ± 5.57 vs. 43.65 ± 5.61, *P*=0.025, Figure 4(a), and 418.37 ± 99.13 vs. 1050.60 ± 207.22, *P*=0.001, Figure 4(b), respectively).

### 3.4. EA Upregulated the Expression of NPS and NPSR Proteins in the Anterior Cingulate Cortex following the C-CPA Model

**Figure 5:**
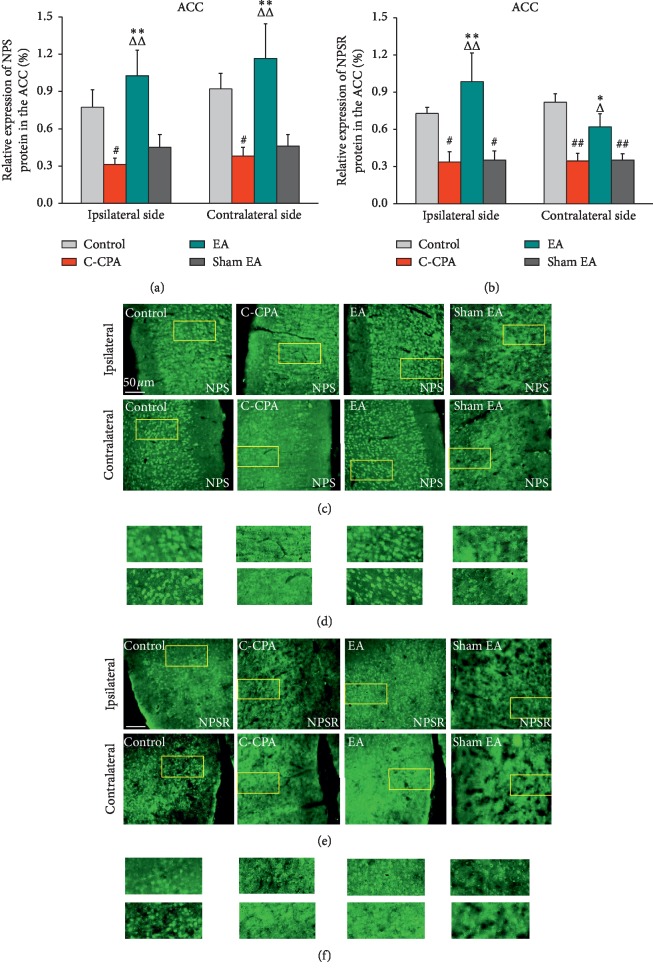
(a) Relative expression of NPS protein (%) in the ACC after EA stimulation in each group. (c) Representative micrographs of bilateral sides (ipsilateral side for the left side and contralateral side for the right side). (b) Relative expression of NPSR protein (%) in the ACC after EA stimulation in each group. (e) Representative micrographs of bilateral sides (ipsilateral side for the left side and contralateral side for the right side). (d, f) High-magnification image of the areas indicated by the yellow squares in (c) and (e). Five rats in each group. ^#^*P* < 0.05 and ^##^*P* < 0.01, C-CPA and sham EA vs. control; ^Δ^*P* < 0.05 and ^ΔΔ^*P* < 0.01, EA vs. C-CPA; ^*∗*^*P* < 0.05 and ^*∗∗*^*P* < 0.01, EA vs. sham EA.

We observed C-CPA leads to downregulation of NPS protein on both sides in the ACC (ipsilateral: 0.3120 ± 0.0505 vs. 0.7740 ± 0.1415, *P*=0.031; contralateral: 0.3800 ± 0.0693 vs. 0.9220 ± 0.1235, *P*=0.033, Figure 5(a)) compared with the control group. The results demonstrated that the NPS expression was significantly elevated bilaterally in the EA group (ipsilateral: 1.0260 ± 0.2070 vs. 0.3120 ± 0.0505, *P*=0.002; contralateral: 1.166 ± 0.2804 vs. 0.3800 ± 0.0693, *P*=0.004, Figure 5(a)). Sham EA made no effect on the expression of NPS protein in the C-CPA model (Figure 5(a)).

As for the NPSR expression, the results were similar to those of the NPS expression in the ACC. The C-CPA model remarkably decreased the expression of NPSR protein on both sides of the ACC (ipsilateral: 0.3375 ± 0.0837 vs. 0.7280 ± 0.0490, *P*=0.035; contralateral: 0.3450 ± 0.0625 vs. 0.8180 ± 0.0693, *P* ≤ 0.001, Figure 5(b)). The NPSR expression in the EA group was explicitly higher than that in the model group (ipsilateral: 0.9850 ± 0.2289 vs. 0.3375 ± 0.0837, *P*=0.003; contralateral: 0.6200 ± 0.1060 vs. 0.3450 ± 0.0625, *P*=0.024, Figure 5(b)). Conversely, sham EA did not show any effect on the expression of NPSR protein of the model.

### 3.5. EA Upregulated the Expression of NPS and NPSR Proteins in the Hypothalamus following the C-CPA Model

**Figure 6:**
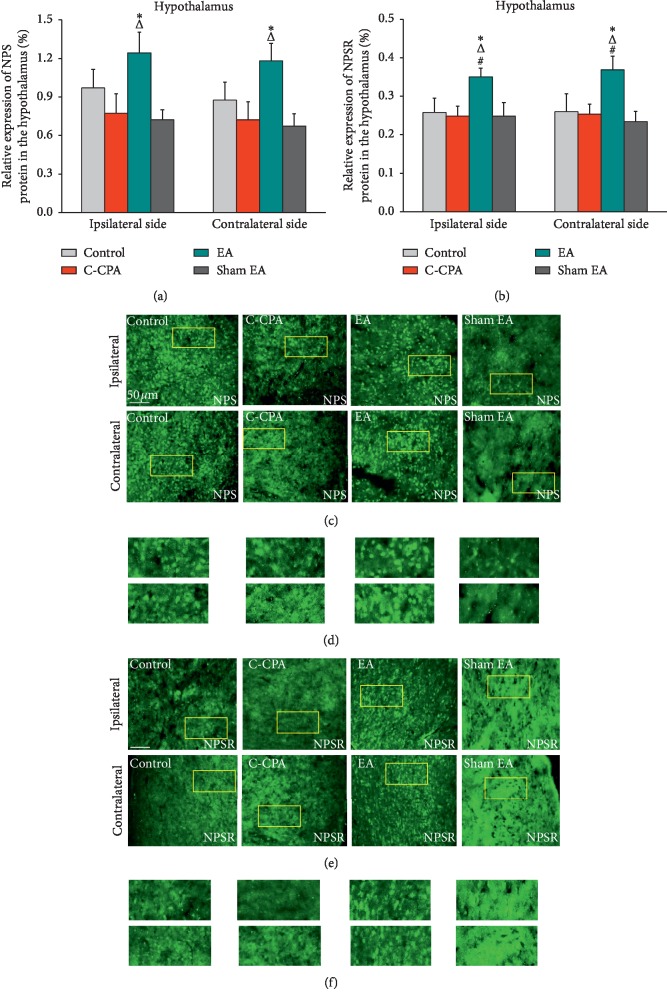
(a) Relative expression of NPS protein (%) in the hypothalamus after EA stimulation in each group. (c) Representative micrographs of bilateral sides (ipsilateral side for the left side and contralateral side for the right side). (b) Relative expression of NPSR protein (%) in the hypothalamus after EA stimulation in each group. (e) Representative micrographs of bilateral sides (ipsilateral side for the left side and contralateral side for the right side). (d, f) High-magnification image of the areas indicated by the yellow squares in (c) and (e). Five rats in each group. ^#^*P* < 0.05, EA vs. control; ^Δ^*P* < 0.05, EA vs. C-CPA; ^*∗*^*P* < 0.05, EA vs. sham EA.

Results were a little different in the hypothalamus; that is, it could not be directly seen from Figure 6(a) that the pain-related emotion evoked the downregulation of the NPS expression, but surprisingly, EA upregulated the NPS expression when compared with the model group (ipsilateral: 1.2429 ± 0.1624 vs. 0.7729 ± 0.1503, *P*=0.024; contralateral: 1.1814 ± 0.1345 vs. 0.7214 ± 0.1403, *P*=0.017, Figure 6(a)), as well as in comparison with the sham EA group (ipsilateral: 1.2429 ± 0.1624 vs. 0.7220 ± 0.0791, *P*=0.023; contralateral: 1.1814 ± 0.1345 vs. 0.6720 ± 0.0974, *P*=0.016, Figure 6(a)).

We next evaluated the expression of NPSR protein in the hypothalamus. Parallel to the findings on the NPS expression, the C-CPA model did not cause prominent downregulation of the NPSR expression (Figure 6(b)). However, EA produced some effects on this protein as it prompted the increase of the NPSR expression in this nucleus bilaterally in comparison with the model group (ipsilateral: 0.3500 ± 0.0225 vs. 0.2483 ± 0.0259, *P*=0.21; contralateral: 0.3683 ± 0.0356 vs. 0.2533 ± 0.0255, *P*=0.22, Figure 6(b)). Furthermore, the EA group held a higher NPSR protein level than the control group (ipsilateral: 0.3500 ± 0.0225 vs. 0.2580 ± 0.0365, *P*=0.042; contralateral: 0.3683 ± 0.0356 vs. 0.2600 ± 0.0460, *P*=0.038, Figure 6(b)). Sham EA failed to increase the NPSR expression in this nucleus bilaterally.

### 3.6. EA Upregulated the Expression of NPSR Specifically in the Ipsilateral Central Amygdala, but Not the NPS Expression, following the C-CPA Model

**Figure 7:**
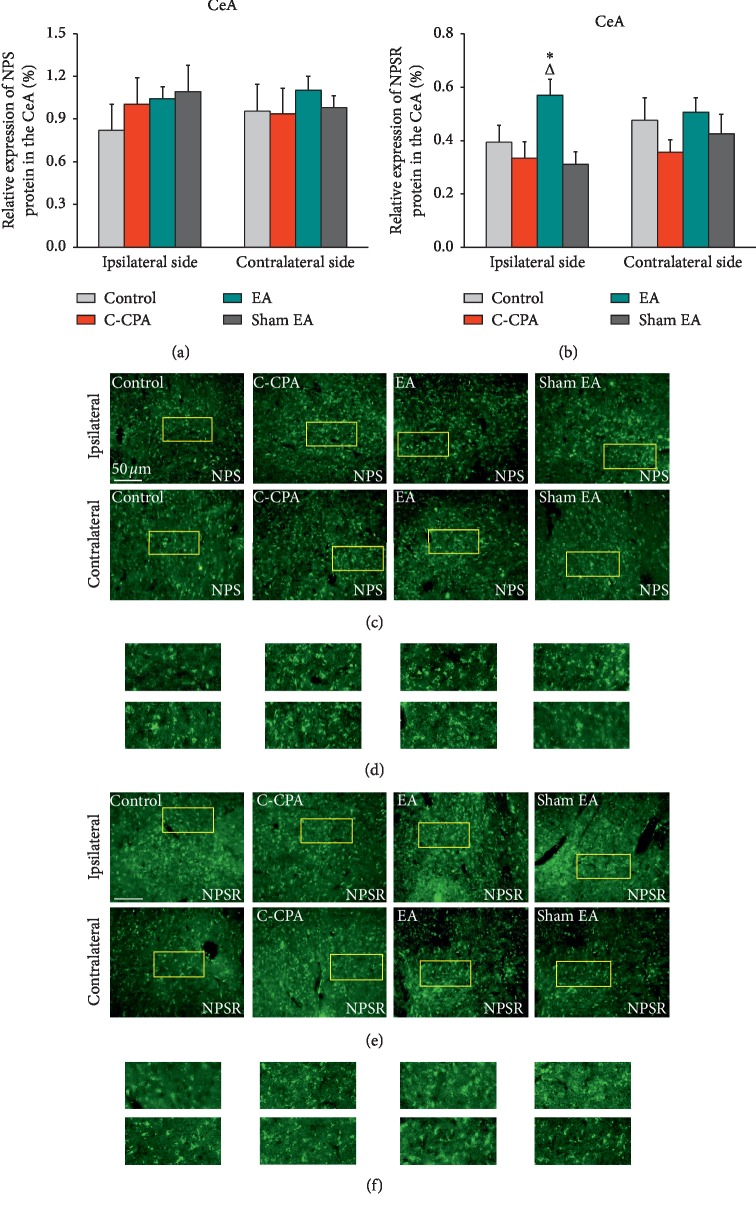
(a) Relative expression of NPS protein (%) in the CeA after EA stimulation in each group. (c) Representative micrographs of bilateral sides (ipsilateral side for the left side and contralateral side for the right side). (b) Relative expression of NPSR protein (%) in the CeA after EA stimulation in each group. (e) Representative micrographs of bilateral sides (ipsilateral side for the left side and contralateral side for the right side). (d, f) High-magnification image of the areas indicated by the yellow squares in (c) and (e). Five rats in each group. ^Δ^*P* < 0.05, EA vs. C-CPA; ^*∗*^*P* < 0.05, EA vs. sham EA.

Here, in our study, we did not find any strong evidence that the C-CPA model affected the NPS expression in the CeA in CFA-injected rats, and there was no difference in this protein level among groups (Figure 7(a)).

Unlike the discovery found in the NPS protein expression in this nucleus, the NPSR protein expression was found to have an increase in EA-treated rats. As demonstrated in Figure 7(b), EA at least prospered the NPSR protein expression in the ipsilateral side of the CeA (0.5690 ± 0.0603 vs. 0.3340 ± 0.0611, *P*=0.013, Figure 7(b)). No positive findings were detected in the sham EA group (Figure 7(b)). A little difference was observed in the contralateral side among groups (Figure 7(b)).

### 3.7. EA Upregulated the Expression of NPS mRNA in the Anterior Cingulate Cortex, but Not the NPSR mRNA Expression, following the C-CPA Model

**Figure 8:**
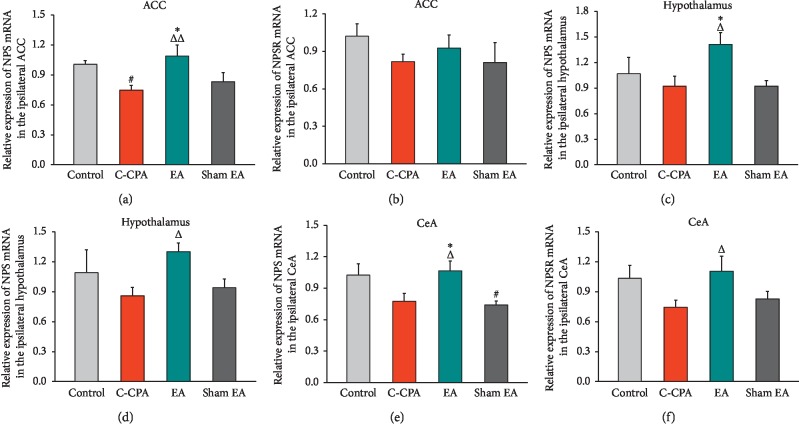
Relative expression of (a) NPS mRNA and (b) NPSR mRNA in the ipsilateral ACC after EA stimulation in each group; (c) NPS mRNA and (d) NPSR mRNA in the ipsilateral hypothalamus after EA stimulation in each group; and (e) NPS mRNA and (f) NPSR mRNA in the ipsilateral CeA after EA stimulation in each group. Five rats in each group. ^#^*P* < 0.05, C-CPA and sham EA vs. control; ^Δ^*P* < 0.05 and ^ΔΔ^*P* < 0.01, EA vs. C-CPA; ^*∗*^*P* < 0.05, EA vs. sham EA.

Since EA stimulation could regulate the protein expression of this system in the ACC, the regulating effect of EA on this system at the genetic level was assessed by real-time PCR. Compared with the control group, the C-CPA model reduced the level of the NPS mRNA expression, as shown by a significant difference between the two groups (1.006 ± 0.035 vs. 0.7460 ± 0.467, *P*=0.033, Figure 8(a)). EA intervention dramatically upregulated the level of NPS mRNA in comparison with the model group (1.0880 ± 0.113 vs. 0.7460 ± 0.467, *P*=0.007, Figure 8(a)). Sham EA displayed no effect on NPS and NPSR mRNA expressions.

In contrast, the NPSR mRNA level was not affected by the C-CPA model, and no difference was observed among groups (Figure 8(b)).

### 3.8. EA Upregulated the Expression of NPS and NPSR mRNA in the Hypothalamus following the C-CPA Model

The results were very similar to those of the protein expression of this system after EA stimulation. C-CPA did not show any impact on the gene expression of this system, but EA administration elicited upregulation of both NPS and NPSR mRNA levels after model establishment (1.412 ± 0.140 vs. 0.920 ± 0.119, *P*=0.022, Figure 8(c); 1.30 ± 0.088 vs. 0.858 ± 0.083, *P*=0.035, Figure 8(d)). Sham EA had no effect on the gene expression when performed on C-CPA.

### 3.9. EA Upregulated the Expression of NPS and NPSR mRNA in the CeA following the C-CPA Model

In our study, the C-CPA model seemed to have no impacts on the fluorescent expression of this system in this region and EA displayed weak regulating effects on this model at the protein level. Here, real-time PCR showed different findings. C-CPA also failed to affect the gene expression of this system. However, an upregulation of NPS and NPSR mRNA was detected after EA stimulation in contrast to the model group (1.066 ± 0.0960 vs. 0.774 ± 0.075, *P*=0.026, Figure 8(e); 1.104 ± 0.153 vs. 0.744 ± 0.072, *P*=0.039, Figure 8(f)). Sham EA produced no therapeutic effect on this model.

## 4. Discussion

A persistent hyperalgesia model induced by CFA has been introduced to mimic peripheral tissue inflammation in rodents parallel to clinical chronic inflammatory pain in the last decades. CPA was used widely as a sensitive test to evaluate the aversive motivational states during chronic opioid withdrawal. Recently, this paradigm has been successfully exploited to assess the aversion state induced by various noxious stimuli. Previous studies have reported the combination of CFA injection with the CPA paradigm (C-CPA) to examine inflammatory pain and on this basis to further study the affective dimension of pain and confirmed that CPA induced by nociceptive stimuli could lead to acute maintenance of affective response to pain in the pain-paired compartment among different strings of rodents [32, 45, 46]. Here, we used the C-CPA model as the substrate to verify the establishment of affective pain. The present study showed the decreasing PWTs after CFA injection lasted at least 3 days and the model group spent less time in the pain-paired compartment on day 4, indicating the success on C-CPA model establishment. The CPA scores highlighted aversion intensity in the CFA-injected animals. Our results were consistent with reports focused on CPA induced by formalin injection in that affective pain was inevitably generated on exposure to inflammatory agents [47].

It is well known that pain could cause many psychiatric disorders such as anxiety and depression due to its multiple dimensions. EA has widely been used to treat pain-related disorders including the sensory and affective pain, but the effects of EA on these disorders are less studied. A clinical trial showed that EA treatment improved symptoms in patients suffering from posttraumatic stress disorder (PTSD) [48]. A pilot and controlled trial demonstrated women who received acupuncture after breast cancer surgery experienced a significantly greater reduction in pain and anxiety [49]. In reports similar to ours, the mitigative effects of EA on the C-CPA model were further elucidated [32, 37, 44, 46]. To address the interventional efficacy of EA on the C-CPA model, we gave this model EA treatment from the conditioning day till day 6. We were surprised to discover the persistent affective pain on day 6. We were reasonable to deduce the result originating from the sensory pain state sustaining at least 3 days (Figure 2(a)). In our current study, EA remarkably abolished the aversive response to reach a level corresponding to the preconditioning session and affected the CPA scores (Figures 3(a) and 3(b)). Obviously, EA offered a much better efficacy than sham EA due to subcutaneous suspension without any manipulation. To sum up, our results further suggested affective pain induced by the C-CPA model could last at least 3 days and this negative affection could be inhibited by EA stimulation.

EA has been broadly introduced to alleviate sensory pain and affective pain among clinical cases in the earlier time [50, 51]. However, the mechanisms underlying the effects of EA on pain-related emotion are not completely understood. Prior to our recent study, there were reports which revealed that anxiety-like behaviors derived from various pain states could be measured with the elevated plus maze (EPM) [52, 53]. Here, we used the EOM to record the data including the time spent and the distance traveled in the open environment as well as the total distance traveled on day 6 after EA stimulation. Our results were consistent with the previous studies that anxiety-like behaviors could be identified in the EOM in a pain state and that EA abolished the anxiety to some extent [38, 52, 53]. Here, EA produced an increased time spent and distance traveled in the open arms. The acupoints chosen in our study are common in treating pain and emotional diseases. We assessed the analgesic effects of EA treatment in the first place and the inhibitory effects on negative emotion straight after. EA displayed preponderant efficacy on both dimensions of pain, whereas sham EA had no effects on both states.

NPS, with its receptor NPSR, had initially been validated and expanded to possess comprehensive physiological functions such as antianxiety, analgesia, and arousal [27–29]. Taking characteristics of distribution into consideration, the NPS-NPSR system may have close link with pain-related emotion. NPS precursor mRNA is highly expressed and mainly restricted in the locus coeruleus (LC), the principal trigeminal sensory nucleus (Pr5), and the lateral parabrachial nucleus (LPB), and a small number of NPS-positive neurons are detected in other brain areas like the amygdala and hypothalamus in a rat brain. In contrast, in situ hybridization shows prominent NPSR mRNA expression signals across vast areas of the nervous system, manifested at high levels in the cortex, hypothalamus, and amygdala and to a lesser degree in Cg1 of the ACC [27–29]. By using the tail withdrawal test and hot-plate test in mice, ICV injection of NPS (0.01–1 nmol) induced a significant increase of tail and paw withdrawal latency, indicating the potential antinociception of the peptide [54]. Through the activation of NPSR, NPS could stimulate the monocyte chemotaxis [55], and related neurons synthesize and release NPS to modulate immune responses, especially in inflammatory responses such as asthma, arthritis, and complex local pain syndrome [56]. Furthermore, murine animals express a large amount of NPSR, so this peptidergic system may be involved in the inflammatory phenotype through the neural mediation mechanism [57]. Patients suffering pain syndrome have cognition and memory of pain sensation and pain-related emotion. The inhibitory avoidance (IA) test and object recognition test provided the evidence that the NPS-NPSR system could enhance the recognition memory during consolidation and reduce inhibitory avoidance memory [58], suggesting the involvement in memory formation and aversion generation. As such, we may deduce from the pattern of the NPSR expression that it suggests potential functions in emotional and sensory processing. We also have evidence-based reasons to choose the ACC, hypothalamus, and CeA as the targeted brain regions to explore partial mechanisms since this system [34, 59, 60] was detected being involved in emotional regulation within these areas.

We thereby examined the protein and mRNA expressions of NPS and NPSR in the candidate regions. Double immunofluorescence staining of NPS/NPSR and NeuN/GFAP (see in supplementary data ([Supplementary-material supplementary-material-1])) showed that NPSR was coexpressed with NeuN but not GFAP, indicating that NPSR was expressed on NeuN. The current results showed that the C-CPA model had a certain inhibitory effect on the mRNA and protein levels of NPS in the ACC which were prevented strongly by EA stimulation. The pronounced change that NPS protein started to decrease at a time when its mRNA was declining could explain the observation of the maintained affective pain. It appeared that the upregulation of these two substances occurred in synchrony when EA intervened. We also tested the changes in NPSR protein and mRNA on the EA regulating effect. We demonstrated the C-CPA model evoked the decrease in NPSR at the protein level but not the mRNA level. EA just promoted the NPSR protein expression. It has been reported previously that the mRNA changes are not necessary in correlation with changes in the corresponding proteins due to the complex process on the mapping between mRNA and protein expressions [61]. Notably, the ACC widely connects to areas like the amygdala, hypothalamus, thalamic nuclei, and hippocampus that are in close link to processing regarding pain and affection [62–64]. The role of the ACC in processing sensory and affective pain has been studied in many laboratories [11, 65]. In common with the central nervous system, glutamate and gamma-aminobutyric acid (GABA) dominate in this area. For one thing, the increased presynaptic glutamate release in the ACC neurons was proved to ascribe to the peripheral inflammation and nerve injury [66, 67]. For another thing, the increased GABAergic transmitter release in the ACC facilitated the elevated mechanical threshold [68]. Clinical reports also suggested a GABA deficit exists in the ACC among patients suffering psychiatric or panic disorders [62, 63] and an acute enhancement of GABAergic activity during antidepressant treatment [69]. NPS activates G*α*_q_ and G*α*_s_ signaling pathways via NPSR inducing mobilization of Ca^2+^ and activating accumulation of cAMP to exert its functions [27, 29]. In the meanwhile, we learned from other studies that the NPS precursor is coexpressed with glutamate in the brainstem [27], and a corresponding NPSR1 gene variation showed that NPSR potentially modulates glutamatergic activity in the ACC to increase the risk for panic disorder [70]. In conclusion, the NPS-NPSR system seems interested in involving in GABA and glutamate neurotransmission to process negative emotion. As described above, we perorated that EA upregulated the expression of NPS and NPSR in the ACC to induce a cohort of intracellular signaling cascades to achieve the alleviation of affective pain.

The hypothalamus is known to be involved in multiple brain functions, and the representative ones are hormone synthesis, biological rhythm determination, emotional behavior, and arousal. The hypothalamus has afferent and efferent connections to, for instance, the cerebral cortex, amygdala, thalamus, hippocampus, periaqueductal gray (PAG), and spinal cord implicated in the sensory and affective dimensions of pain [71]. Trials on animal models have proven well the special role of multiple subdivisions of the hypothalamus in affective pain [14, 15]. Central stress-integrative circuits suggested forebrain glutamatergic and GABAergic projects to the dorsomedial hypothalamus (DMH), and microinjection of a GABA_A_ agonist or antagonist seemed like inducing increases or decreases in the social interaction time, respectively [72]. Referential studies demonstrated intracerebroventricular and intraventromedial hypothalamus (iVMH) administration of NPS was capable of increasing rearing and locomotor activity and stimulating the HPA axis [60]. NPS and NPSR were found highly expressed in the hypothalamus [27–29]. Combining with the recognized functions of the NPS-NPSR system in the central nervous system, we assumed EA mediates the alleviation of affective pain through this system within the hypothalamus. The results herein indicated the C-CPA model did not degenerate the protein and mRNA expressions of both NPS and NPSR in the hypothalamus; nevertheless, EA significantly enhanced the expression of the two proteins on both sides as well as the mRNA expression of this system in the hypothalamus compared with the remaining groups. The parallel increases in both dimensions after EA treatment reveal the possible mechanism under EA regulating effects on affective pain. One must also consider that NPS and NPSR are highly expressed in the hypothalamus under normal circumstances. Noxious stimuli in our experiment might not be enough to elicit obvious attenuation of the targets' protein and mRNA expressions. Since the NPS-NPSR system is vigorous in mobilizing the activation of glutamate and GABA transmission, it is thus speculated EA stimulation might arrive at the hypothalamus to reinforce the mRNA and protein expressions of this system and in turn activates a strand of cellular signaling pathways acting on the descending pathway including the PAG, a crucial structure in the endogenous descending inhibitory system, to participate in the encoding and processing of affective pain.

Although the NPS precursor expression appeared as a sparse and scattered pattern in the amygdala complex, we still found the existence of NPS protein in the subdivision of the amygdala which was not separately put forward by initial reports. Speaking of the NPSR expression in this district, a recent finding confirmed the NPSR mRNA expression in the CeA. Earlier studies detected no NPSR mRNA expression in this area but did detect the signal in the lateral nucleus of the amygdala (LA), the basolateral nucleus of the amygdala (BLA), etc. [27, 29, 73]. The interesting discovery in our report was the significant increase in the NPSR expression on the ipsilateral side alone after EA stimulation. Reports for reference on the inner link between the endogenous NPS-NPSR system and the amygdala regarding affective pain predominantly focused on the LA, BLA, or the whole complex. Although there have been studies validating the involvement of the NPS-NPSR system in the CeA under both dimensions during the pain state, almost all chose the exogenous administration of NPS or NPSR as the exploratory method. Along with the change in the protein level, we detected both NPS and NPSR mRNA expressions were upregulated after EA stimulation. Aside from the dispensable prerequisite from mRNA to protein synthesis, the protein expression may lag behind the mRNA expression [61]. Here, in the present report, the C-CPA model failed in deteriorating the expression of the system and EA exerted impotent efficacy because of the insufficient activated amount of the NPS-NPSR system in the CeA neurons or, as a matter of fact, EA per se did not mediate the affective pain regulation at the CeA level through the NPS-NPSR system. Therefore, it still needs further study to verify the involvement of the NPS-NPSR system in this brain region on affective pain alleviation mediated by EA.

## 5. Conclusions

Our current study demonstrated that the pain-related emotion could be caused by the inflammatory stimulus and EA stimulation showed good alleviation effects on affective pain following the chronic pain state. These therapeutic effects on pain-related emotion might be associated with the activation and upregulation of the NPS-NPSR system at protein and mRNA levels in the ACC and hypothalamus. However, whether this similar mechanism occurs in the CeA during EA stimulation on affective pain needs to be further studied.

## Data Availability

All data sets generated and analyzed during the whole study are available from the corresponding author on reasonable request.

## Supplementary Materials

Supplementary Materials Double immunofluorescence staining of NPS (a–c)/NPSR (g–i) and NeuN and NPS (d–f)/NPSR (j–l) and GFAP. (a–c) Coexpression of NPS (green) and NeuN (red) and the merged image. (d–f) Coexpression of NPS (green) and GFAP (red) and the merged image. (g–i) Coexpression of NPSR (green) and NeuN (red) and the merged image. (j–l) Coexpression of NPSR (green) and GFAP (red) and the merged image.
