# Supplementary figures and images for: Electroacupuncture Alleviates Pain-Related Emotion by Upregulating the Expression of NPS and Its Receptor NPSR in the Anterior Cingulate Cortex and Hypothalamus
Source: Evid Based Complement Alternat Med. 2020 Feb 10;2020:8630368. doi: 10.1155/2020/8630368 (PMC7035524; doi:10.1155/2020/8630368)

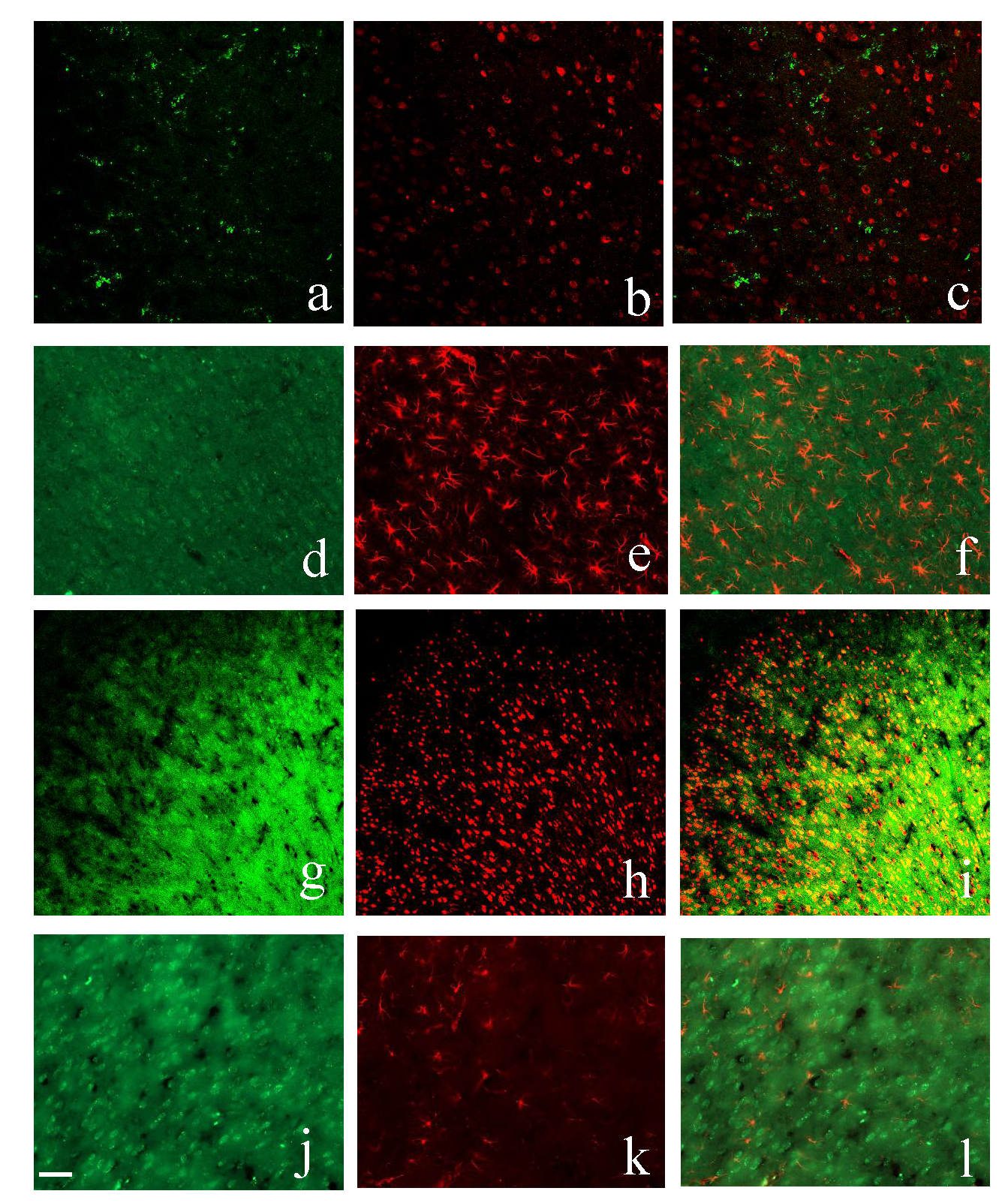

Supplement: Supplementary Materials — Double immunofluorescence staining of NPS (a–c)/NPSR (g–i) and NeuN and NPS (d–f)/NPSR (j–l) and GFAP. (a–c) Coexpression of NPS (green) and NeuN (red) and the merged image. (d–f) Coexpression of NPS (green) and GFAP (red) and the merged image. (g–i) Coexpression of NPSR (green) and NeuN (red) and the merged image. (j–l) Coexpression of NPSR (green) and GFAP (red) and the merged image. [file 8630368.f1.tiff]
